# Complete genome sequence of the halophilic and highly halotolerant *Chromohalobacter salexigens* type strain (1H11^T^)

**DOI:** 10.4056/sigs.2285059

**Published:** 2011-12-30

**Authors:** Alex Copeland, Kathleen O’Connor, Susan Lucas, Alla Lapidus, Kerrie W. Berry, John C. Detter, Tijana Glavina Del Rio, Nancy Hammon, Eileen Dalin, Hope Tice, Sam Pitluck, David Bruce, Lynne Goodwin, Cliff Han, Roxanne Tapia, Elizabeth Saunders, Jeremy Schmutz, Thomas Brettin, Frank Larimer, Miriam Land, Loren Hauser, Carmen Vargas, Joaquin J. Nieto, Nikos C. Kyrpides, Natalia Ivanova, Markus Göker, Hans-Peter Klenk, Laszlo N. Csonka, Tanja Woyke

**Affiliations:** 1 DOE Joint Genome Institute, Walnut Creek, California, USA; 2 Department of Biological Sciences, Purdue University, West Lafayette, Indiana, USA; 3 Los Alamos National Laboratory, Bioscience Division, Los Alamos, New Mexico, USA; 4 Oak Ridge National Laboratory, Oak Ridge, Tennessee, USA; 5 Department of Microbiology and Parasitology, University of Seville, Spain; 6 Leibniz Institute DSMZ – German Collection of Microorganisms and Cell Cultures, Braunschweig, Germany

**Keywords:** aerobic, chemoorganotrophic, Gram-negative, motile, moderately halophilic, halo tolerant, ectoine synthesis, *Halomonadaceae*, *Gammaproteobacteria*, DOEM 2004

## Abstract

*Chromohalobacter salexigens* is one of nine currently known species of the genus *Chromohalobacter* in the family *Halomonadaceae*. It is the most halotolerant of the so-called ‘moderately halophilic bacteria’ currently known and, due to its strong euryhaline phenotype, it is an established model organism for prokaryotic osmoadaptation. *C. salexigens* strain 1H11^T^ and *Halomonas elongata* are the first and the second members of the family *Halomonadaceae* with a completely sequenced genome. The 3,696,649 bp long chromosome with a total of 3,319 protein-coding and 93 RNA genes was sequenced as part of the DOE Joint Genome Institute Program DOEM 2004.

## Introduction

Strain 1H11^T^ (= DSM 3043 = ATCC BAA-138 = CECT 5384) is the type strain of the species *Chromohalobacter salexigens* [1], which is one of currently nine species in the genus *Chromohalobacter* [1,2]. The genus name was derived from the Greek words *chroma*, color, *hals halos*, salt, and the Neo-Latin *bacter*, rod, meaning the colored salt rod. The species epithet originated from the Latin words *sal salis*, salt, and *exigo*, to demand; salt-demanding [3]. Strain 1H11^T^ was originally isolated in 1974 in Bonair, Netherlands Antilles, from salterns containing 18.6% salt, and was initially published as a strain belonging to the species *Halomonas elongata* [4]. In 2001, Arahal *et al*. transferred the strain to the genus *Chromohalobacter* [2] as the type strain of the then novel species *C. salexigens* [1] following detailed phenotypic, genotypic, and phylogenetic analyses. *C. salexigens* is known for its very broad salinity range [1] and for its role as a model organism for prokaryotic osmosadaptation [5-7], e.g. the synthesis of ectoines (ectoine and hydroxyectoine) for cell stress protection [8,9]. Here we present a summary classification and characteristics of *C. salexigens* 1H11^T^, together with the description of the complete genomic sequencing and annotation.

## Classification and features

The sequences of the five identical 16S rRNA genes of strain 1H11^T^ were compared using NCBI BLAST [10] under default settings (e.g., considering only the high-scoring segment pairs (HSPs) from the best 250 hits) with the most recent release of the Greengenes database [11] and the relative frequencies of taxa and keywords (reduced to their stem [12]) were determined and weighted by BLAST scores. The most frequently occurring genera were *Halomonas* (50.7%), *Chromohalobacter* (46.3%), '*Haererehalobacter*' (1.7%), *Bacillus* (0.8%) and *Pseudomonas* (0.5%) (214 hits in total). For 16 hits to sequences from members of the *C. salexigens* species, the average identity within HSPs was 99.9% and the average coverage by HSPs was 97.9%. For 22 hits to sequences from other members of the genus *Chromohalobacter*, the average identity within HSPs was 98.2% and the average coverage by HSPs was 98.6%. Among all other species, the one yielding the highest score was *Chromohalobacter marismortui* (X87222), which corresponded to an identity of 99.9% and an HSP coverage of 100.0%. (Note that the Greengenes database uses the INSDC (= EMBL/NCBI/DDBJ) annotation, which is not an authoritative source for nomenclature or classification.) The highest-scoring environmental sequence was EU799899 ('It's all ranking aquatic Newport Harbor RI clone 1C227569'), which showed an identity of 100.0% and an HSP coverage of 100.0%. The most frequently occurring keywords within the labels of environmental samples which yielded hits were 'soil' (12.1%), 'lake' (3.6%), 'salin' (3.0%), 'agricultur' (2.9%) and 'alkalin, chang, flood, former, mexico, texcoco' (2.6%) (36 hits in total). The most frequently occurring keyword within the labels of environmental samples which yielded hits of a higher score than the highest scoring species was 'aquat, harbour, newport, rank' (25.0%) (2 hits in total). These keywords fit reasonably well with the ecological and physiological properties reported for strain 1H11^T^ in the original description [1].

**Figure 1:**
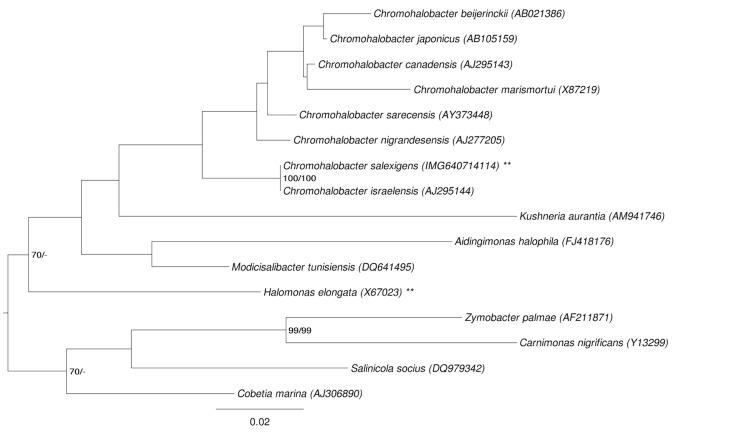
Phylogenetic tree highlighting the position of *C. salexigens* relative to the type strains of the other species within the genus and the type species of the other genera within the family *Halomonadaceae*. The tree was inferred from 1,440 aligned characters [13,14] of the 16S rRNA gene sequence under the maximum likelihood (ML) criterion [15]. Rooting was done initially using the midpoint method [16] and then checked for its agreement with the current classification (Table 1). The branches are scaled in terms of the expected number of substitutions per site. Numbers adjacent to the branches are support values from 1,000 ML bootstrap replicates [17] (left) and from 1,000 maximum parsimony bootstrap replicates [18] (right) if larger than 60%. Lineages with type strain genome sequencing projects registered in GOLD [19] are labeled with one asterisk, those also listed as 'Complete and Published' with two asterisks [20].

Figure 1 shows the phylogenetic neighborhood of *C. salexigens* in a 16S rRNA based tree. The sequences of the five identical 16S rRNA gene copies in the genome differ by two nucleotides from the previously published 16S rRNA sequence (AJ295146), which contains three ambiguous base calls.

**Table 1:** Classification and general features of *C. salexigens* according to the MIGS recommendations [21].

**MIGS ID**	**Property**	**Term**	**Evidence code**
	Current classification	Domain *Bacteria*	TAS [22]
		Phylum *Proteobacteria*	TAS [23]
		Class *Gammaproteobacteria*	TAS [24,25]
		Order *Oceanospirillales*	TAS [24,26]
		Family *Halomonadaceae*	TAS [27-31]
		Genus *Chromohalobacter*	TAS [2,32]
		Species *Chromohalobacter salexigens*	TAS [1]
		Type strain 1H11	TAS [1,4]
	Gram stain	negative	TAS [1]
	Cell shape	rod-shaped	TAS [1]
	Motility	motile	TAS [1]
	Sporulation	none	TAS [1]
	Temperature range	mesophilic, 15–45°C	TAS [1]
	Optimum temperature	37°C	TAS [1]
	Salinity	halophilic and halotolerant. Salinity range from 0.9 to 32% (w/v) NaCl in rich media, 2.9% to 19% (0.5 M to 3.75M) NaCl in minimal media; halotolerance increased by osmoprotectants; halotolerance decreases at high temperature.	TAS [1,4,6,33]
MIGS-22	Oxygen requirement	uses O_2_ and NO_3_^-^ as electron acceptors; does not grow fermentatively	TAS [4]
	Carbon source	various organic acids, alcohols, sugars, and aromatic compounds	TAS [1]
	Energy metabolism	chemoorganotrophic	NAS
MIGS-6	Habitat	saltern, fresh water	TAS [4]
MIGS-15	Biotic relationship	free living	TAS [1]
MIGS-14	Pathogenicity	none	NAS
	Biosafety level	1	TAS [34]
	Isolation	solar salt facility, concentration more than 10% NaCl	TAS [1]
MIGS-4	Geographic location	Bonaire, Netherlands Antilles	TAS [1]
MIGS-5	Sample collection time	June 1974	TAS [4]
MIGS-4.1	Latitude	12.25	NAS
MIGS-4.2	Longitude	-68.26	NAS
MIGS-4.3	Depth	surface	NAS
MIGS-4.4	Altitude	sea level	NAS

Evidence codes - TAS: Traceable Author Statement (i.e. a direct report exists in the literature); NAS: Non-traceable Author Statement (i.e. not directly observed for the living, isolated sample, but based on a generally accepted property for the species, or anecdotal evidence). These evidence codes are from of the Gene Ontology project [35].

**Figure 2:**
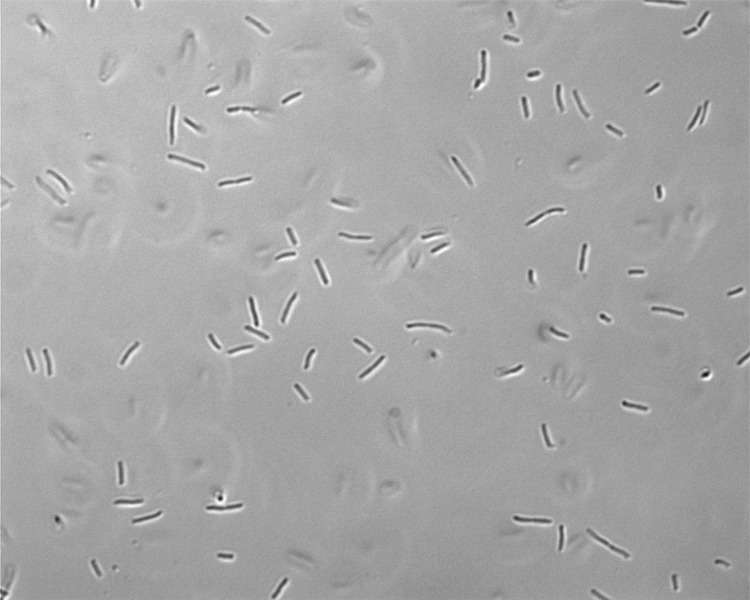
Light microscopic image of *C. salexigens* 1H11^T^

Cells of *C. salexigens* strain 1H11^T^ are straight or slightly curved rods, 0.7 to 1.0 by 2 to 3 µm in size (Figure 2) with squared ends and occur singly or in pairs [1,4]. Cells of strain 1H11^T^ stain Gram-negative, are motile with polar flagella, strictly aerobic, and are non-spore-forming [1,4]. Carbon and nitrogen source utilization and biochemistry of the strain were reported by Arahal *et al*. [1]. A partial characterization of the carbon-source utilization by the organism has also been presented by Csonka *et al.* [36], who reported that the strain can degrade a number of aromatic compounds, including benzoate, protocatechuate, 4-hydroxybenzoate, and toluene.

*C. salexigens* 1H11^T^ is a halophile, which according to the classification proposed by Kushner [37], is on the borderline between "moderate" halophiles (those growing optimally between 2.9 -14.5% NaCl) and "extreme" halophiles (those growing optimally between 8.7 -23.2% NaCl). In addition, it displays extraordinarily high halotolerance (considered as the ability to live and survive under high salt concentrations), and is able to grow at salt concentrations over 17.4% and 32% in defined and complex media, respectively. However, both the minimum NaCl requirement and the upper limit of NaCl tolerance are dependent on growth medium and temperature. The organism can tolerate higher NaCl concentrations in LB or in other complex media than in defined media. In defined media, halotolerance is enhanced by osmoprotectants, such as glycine betaine or its precursor, choline [4,6,33]. In the complex medium SW (‘sea water’), which is routinely used for growing this type of microorganism, strain 1H11^T^ grows optimally at 7.5 to 10% (w/v) NaCl, with growth occurring over the range of 0.9% to ­25% NaCl [1]. In casein medium, which was initially used for strain isolation, growth occurs in the presence of 32% solar salts [4]. In SW medium containing 10% (w/v) total salts, *C. salexigens* 1H11^T^ can grow at a pH range from 5 to 10, with an optimum at pH 7.5 [1]. In the same medium, the temperature range for growth is 15 – 45°C, with an optimum at 37°C [1]. In the standard defined medium M63, supplemented with glucose as the sole carbon source, growth is optimal at 8.7 to 11.6% NaCl but occurs over the range of 2.9% NaCl or a maximum of 19% NaCl [6]. Interestingly, *C. salexigens* 1H11^T^ exhibits maximal growth rate in glucose-M63 with only 1.8% (0.3M) NaCl in the presence of high concentrations of salts of other inorganic ions, including K^+^, Rb^+^, NH_4_^+^, Br^-^, NO_3_^-^, or SO_4_^-^ [38]. However, it is an open question whether this strain is unique among halophiles in being able to use other inorganic ions in addition to Na^+^ and Cl^-^ for maximal growth rate.

### Chemotaxonomy

Data on the structure of the cell wall, fatty acids lipid composition, quinones and polar lipids are not available.

## Genome sequencing and annotation

### Genome project history

This organism was selected for sequencing on the basis of the DOE Joint Genome Institute Program DOEM 2004. The genome project is deposited in the Genomes On Line Database [19] and the complete genome sequence is deposited in GenBank. Sequencing, finishing and annotation were performed by the DOE Joint Genome Institute (JGI). A summary of the project information is shown in Table 2.

**Table 2:** Genome sequencing project information

**MIGS ID**	**Property**	**Term**
MIGS-31	Finishing quality	Finished
MIGS-28	Libraries used	Three genomic Sanger libraries: 4 kb pUC, 8kb pMCL200 and fosmid pcc1Fos libraries.
MIGS-29	Sequencing platforms	ABI3730
MIGS-31.2	Sequencing coverage	11.5 × Sanger
MIGS-30	Assemblers	Phrap
MIGS-32	Gene calling method	Critica complemented with the output of Glimmer
	INSDC ID	CP000285
	GenBank Date of Release	April 16, 2006
	GOLD ID	Gc00371
	NCBI project ID	12636
	Database: IMG	637000075
MIGS-13	Source material identifier	DSM 3043
	Project relevance	Bioremediation, Biotechnology, Environmental

### Strain history

The history of strain 1H11^T^ begins with R.H. Vreeland, who deposited the organism in the DSMZ open collection, where cultures of the strain are maintained freeze dried as well as in liquid nitrogen (since 1984). The strain used for the project was provided by the Carmen Vargas – Joaquín Nieto lab in Seville (Spain), who acquired it from the DSMZ.

### Growth conditions and DNA isolation

The culture of strain 1H11^T^, DSM 3043, used to prepare genomic DNA (gDNA) for sequencing was grown in LB medium with 1 M NaCl. DNA was extracted as described by O’Connor and Zusman [39]. The purity, quality and size of the bulk gDNA preparation were assessed by JGI according to DOE-JGI guidelines.

### Genome sequencing and assembly

The genome was sequenced using a combination of 4 kb, 8 kb and fosmid DNA libraries. All general aspects of library construction and sequencing can be found at the JGI website [40]. Draft assemblies were based on 44,750 total reads. The Phred/Phrap/Consed software package was used for sequence assembly and quality assessment [41]. After the shotgun stage, reads were assembled with parallel phrap (High Performance Software, LLC). Possible mis-assemblies were corrected with Dupfinisher or transposon bombing of bridging clones (Epicentre Biotechnologies, Madison, WI) [42]. Gaps between contigs were closed by editing in Consed, custom priming, or PCR amplification (Roche Applied Science, Indianapolis, IN). A total of 920 additional reactions, 14 shatter and 18 transposon bomb libraries were needed to close gaps and to raise the quality of the finished sequence. The error rate of the completed genome sequence is less than 1 in 100,000. Together all libraries provided 11.5 × coverage of the genome.

### Genome annotation

Genes were identified using two gene modeling programs, Glimmer [43] and Critica [44] as part of the Oak Ridge National Laboratory genome annotation pipeline. The two sets of gene calls were combined using Critica as the preferred start call for genes with the same stop codon. Genes specifying fewer than 80 amino acids that were predicted by only one of the gene callers and had no Blast hit in the KEGG database at ≤1e-05, were deleted. Automated annotation was followed by a round of manual curation to eliminate obvious overlaps. The predicted CDSs were translated and used to search the National Center for Biotechnology Information (NCBI) non-redundant database, UniProt, TIGRFam, Pfam, PRIAM, KEGG, COG, and InterPro databases. These data sources were combined to assert a product description for each predicted protein. Non-coding genes and miscellaneous features were predicted using tRNAscan-SE [45], TMHMM [46], and signalP [47].

## Genome properties

**Figure 3:**
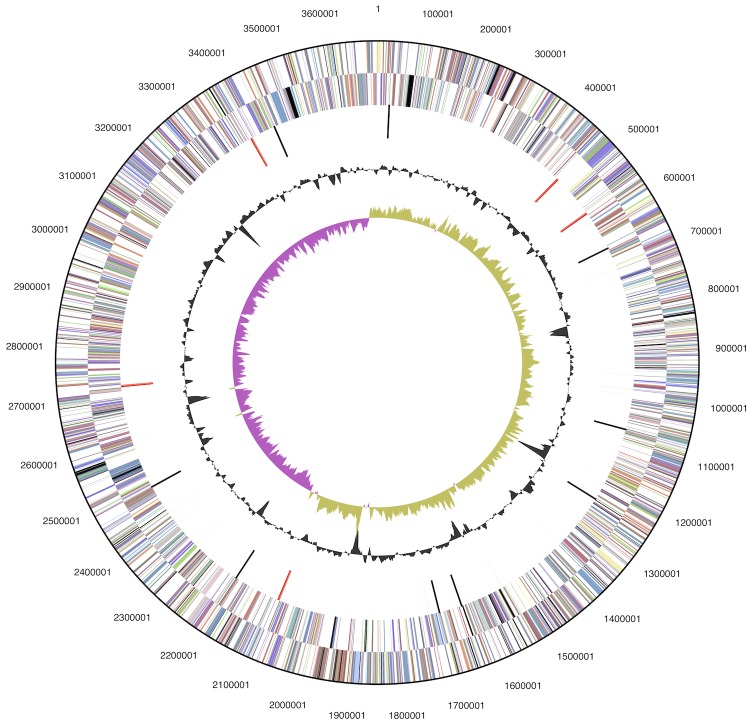
Graphical circular map of the genome. From outside to the center: Genes on forward strand (color by COG categories), Genes on reverse strand (color by COG categories), RNA genes (tRNAs green, rRNAs red, other RNAs black), GC content, GC skew.

The genome consists of a 3,696,649 bp long chromosome with a 63.9% G+C content (Table 3 and Figure 3). Of the 3,412 putative genes, 3,319 are protein-coding, and 93 specify RNAs; 21 pseudogenes were also identified. The majority of the protein-coding genes (76.8%) were assigned a putative function while the remaining ones were annotated as encoding hypothetical proteins. The distribution of genes into COGs functional categories is presented in Table 4.

**Table 3:** Genome Statistics

**Attribute**	**Value**	**% of Total**
Genome size (bp)	3,696,649	100.00%
DNA coding region (bp)	3,333,410	90.17%
DNA G+C content (bp)	2,362,597	63.91%
Number of replicons	1	
Extrachromosomal elements	0	
Total genes	3,412	100.00%
RNA genes	93	2.73%
rRNA operons	5	
Protein-coding genes	3,319	97.27%
Pseudogenes	21	0.62%
Genes with function prediction	2,621	76.82%
Genes in paralog clusters	402	11.78%
Genes assigned to COGs	2,842	83.29%
Genes assigned Pfam domains	2,928	85.81%
Genes with signal peptides	689	20.19%
Genes with transmembrane helices	828	24.27%
CRISPR repeats	5	

**Table 4:** Number of genes associated with the general COG functional categories

**Code**	**value**	**%age**	**Description**
J	166	5.2	Translation, ribosomal structure and biogenesis
A	1	0.0	RNA processing and modification
K	251	7.8	Transcription
L	114	3.5	Replication, recombination and repair
B	1	0.0	Chromatin structure and dynamics
D	34	1.1	Cell cycle control, cell division, chromosome partitioning
Y	0	0.0	Nuclear structure
V	33	1.0	Defense mechanisms
T	152	4.7	Signal transduction mechanisms
M	184	5.7	Cell wall/membrane biogenesis
N	81	2.5	Cell motility
Z	0	0.0	Cytoskeleton
W	0	0.0	Extracellular structures
U	77	2.4	Intracellular trafficking and secretion, and vesicular transport
O	122	3.8	Posttranslational modification, protein turnover, chaperones
C	207	6.4	Energy production and conversion
G	227	7.1	Carbohydrate transport and metabolism
E	324	10.1	Amino acid transport and metabolism
F	81	2.5	Nucleotide transport and metabolism
H	152	4.7	Coenzyme transport and metabolism
I	110	3.4	Lipid transport and metabolism
P	175	5.4	Inorganic ion transport and metabolism
Q	76	2.4	Secondary metabolites biosynthesis, transport and catabolism
R	385	12.0	General function prediction only
S	269	8.4	Function unknown
-	570	16.7	Not in COGs

## Insights into the genome

The publication of genome sequence strain 1H11^T^ is preceded by some publications that were based on draft versions of the sequence or on publicly available genome sequence and annotation. Oren *et al.* [48] found that the predicted isoelectric points of periplasmic proteins of *C. salexigens* 1H11^T^ are significantly more acidic than those of orthologous proteins in mesophilic bacteria, and they suggested that this feature may contribute to the halophilic characteristics of 1H11^T^. Analysis of the genomic sequence indicted that the organism has all of the enzymes of the Embden-Meyerhof glycolytic pathway, hexose monophosphate shunt, and TCA cycle but seemed to lack the standard fructose-1,6-bisphosphate phosphatase of the gluconeogenetic pathway [36]. Krejcík *et al.* predicted the isethionate formation from taurine based on the genome sequence [49]. Ates *et al.* recently presented a genome-scale reconstruction of a metabolic network for strain 1H11^T^ focusing on the uptake and accumulation of industrially important organic osmolytes such as ectoine and betaine [5].

## References

[1] Arahal DR García MVargas C Cánovas D Nieto JJ Ventosa A *Chromohalobacter salexigens* sp. nov., a moderately halophilic species that includes *Halomonas elongata* DSM 3043 and ATCC 33174. Int J Syst Evol Microbiol 2001; 51:1457-1462 10.1099/00207713-51-4-1457 11491346

[2] Ventosa A Gutierrez MC Garcia MT Ruiz-Berraquero F Classification of " *Chromobacterium **marismortui*" in a new genus, *Chromohalobacter* gen. nov., as *Chromohalobacter marismortui* comb. nov., nom. rev. Int J Syst Bacteriol 1989; 39:382-386 10.1099/00207713-39-4-382

[3] Euzéby JP List of Bacterial Names with Standing in Nomenclature: a folder available on the Internet. Int J Syst Bacteriol 1997; 47:590-592 10.1099/00207713-47-2-590 9103655

[4] Vreeland RH Litchfield CD Martin EL Elliot E *Halomonas elongata*, a new genus and species of extremely salt-tolerant bacteria. Int J Syst Bacteriol 1980; 30:485-495 10.1099/00207713-30-2-485

[5] Ates Ö Oner ET Arga KY Genome-scale reconstruction of metabolic network for a halophilic extremophile, *Chromobacter salexigens* DSM 3043. BMC Syst Biol 2011; 5:12 10.1186/1752-0509-5-12 21251315 PMC3034673

[6] Cánovas D Vargas C Csonka LN Ventosa A Nieto JJ Osmoprotectants in *Halomonas elongata*:high affinity betaine transport system and choline-betaine pathway. J Bacteriol 1996; 178:7221-7226 10.1128/jb.178.24.7221-7226.1996 PMC178636 8955405

[7] Cánovas D Vargas C Csonka LN Ventosa A Nieto JJ Synthesis of glycine betaine from exogenous choline in the moderately halophilic bacterium *Halomonas elongata* Appl Environ Microbiol 1998; 64:4095-4097 9758852 10.1128/aem.64.10.4095-4097.1998 PMC106611

[8] Pastor JM Salvador M Argandona M Bernal V Reina-Buena M Csonka LN Iborra JL Vargas C Nieto JJ Cánovas M Ectoines in cell stress protection: uses and biotechnological production. Biotechnol Adv 2010; 28:782-801 10.1016/j.biotechadv.2010.06.005 20600783

[9] Calderón MI Vargas C Rojo F Iglesias-Guerra F Csonka LN Ventosa A Nieto JJ Complex regulation of the synthesis of the compatible solute ectoine in the halophilic bacterium *Chromohalobacter salexigens* DSM 3043^T^. Microbiology 2004; 150:3051-3063 10.1099/mic.0.27122-0 15347763

[10] Altschul SF Gish W Miller W Myers EW Lipman DJ Basic local alignment search tool. J Mol Biol 1990; 215:403-410 2231712 10.1016/S0022-2836(05)80360-2

[11] DeSantis TZ Hugenholtz P Larsen N Rojas M Brodie EL Keller K Huber T Dalevi D Hu P Andersen GL Greengenes, a chimera-checked 16S rRNA gene database and workbench compatible with ARB. Appl Environ Microbiol 2006; 72:5069-5072 10.1128/AEM.03006-05 16820507 PMC1489311

[12] Porter MF. An algorithm for suffix stripping. Program: *electronic library and information systems* 1980; **14**:130-137.

[13] Lee C Grasso C Sharlow MF Multiple sequence alignment using partial order graphs. Bioinformatics 2002; 18:452-464 10.1093/bioinformatics/18.3.452 11934745

[14] Castresana J Selection of conserved blocks from multiple alignments for their use in phylogenetic analysis. Mol Biol Evol 2000; 17:540-552 10742046 10.1093/oxfordjournals.molbev.a026334

[15] Stamatakis A Hoover P Rougemont J A rapid bootstrap algorithm for the RAxML web servers. Syst Biol 2008; 57:758-771 10.1080/10635150802429642 18853362

[16] Hess PN De Moraes Russo CA An empirical test of the midpoint rooting method. Biol J Linn Soc Lond 2007; 92:669-674 10.1111/j.1095-8312.2007.00864.x PMC7110036 32287391

[17] Pattengale ND Alipour M Bininda-Emonds ORP Moret BME Stamatakis A How many bootstrap replicates are necessary? Lect Notes Comput Sci 2009; 5541:184-200 10.1007/978-3-642-02008-7_13 20377449

[18] Swofford DL. PAUP*: Phylogenetic Analysis Using Parsimony (*and Other Methods), Version 4.0 b10. Sinauer Associates, Sunderland, 2002.

[19] Liolios K Chen IM Mavromatis K Tavernarakis N Kyrpides NC The genomes on line database (GOLD) in 2009: Status of genomic and metagenomic projects and their associated metadata. Nucleic Acids Res 2010; 38:D346-D354 10.1093/nar/gkp848 19914934 PMC2808860

[20] Schwibbert K, Marin-Sanguino A, Bagyan I, Heidrich G, Lentzen G, Seitz H, Rampp M, Schuster SC, Klenk HP, Pfeiffer F, Oesterhelt D, Kunte HJ. A blueprint of ectoine metabolism from the genome of the industrial producer *Halomonas elongata* DSM 2581^T^ *Environ Microbiol* 2010; **13**:1973-1994. 10.1111/j.1462-2920.2010.02336.x PMC3187862 20849449

[21] Field D Garrity G Gray T Morrison N Selengut J Sterk P Tatusova T Thomson N Allen MJ Angiuoli SV The minimum information about a genome sequence (MIGS) specification. Nat Biotechnol 2008; 26:541-547 10.1038/nbt1360 18464787 PMC2409278

[22] Woese CR Kandler O Wheelis ML Towards a natural system of organisms. Proposal for the domains *Archaea* and *Bacteria* Proc Natl Acad Sci USA 1990; 87:4576-4579 10.1073/pnas.87.12.4576 PMC54159 2112744

[23] Garrity GM, Bell JA, Lilburn T. Phylum XIV. *Proteobacteria* phyl. nov. *In*: Brenner DJ, Krieg NR, Staley JT, Garrity GM (*eds*), Bergey's Manual of Systematic Bacteriology, second edition, vol. 2 (The *Proteobacteria*), part B (The *Gammaproteobacteria*), Springer, New York, 2005, p. 1.

[24] Garrity GM, Bell JA, Lilburn T. Class III. *Gammaproteobacteria* class. nov. *In*: Garrity GM, Brenner DJ, Krieg NR, Staley JT (*eds*), Bergey's Manual of Systematic Bacteriology, Second Edition, Volume 2, Part B, Springer, New York, 2005, p. 1.

[25] Validation List 106. Int J Syst Evol Microbiol 2005; 55:2235-2238 10.1099/ijs.0.64108-0

[26] Garrity GM, Bell JA, Lilburn T. Order VIII. *Oceanospirillales* ord. nov. *In:* Garrity GM, Brenner DJ, Krieg NR, Staley JT (*eds*), Bergey's Manual of Systematic Bacteriology, Second Edition, Volume 2, Part B, Springer, New York, 2005, p. 270.

[27] Franzman PD Wehmeyer U Stackebrandt E *Halomonadaceae* fam. nov., a new family of the class *Proteobacteria* to accommodate the genera *Halomonas* and *Deleya* Syst Appl Microbiol 1988; 11:16-19

[28] Validation List No 29. Int J Syst Bacteriol 1989; 39:205-206 10.1099/00207713-39-2-205

[29] Dobson SJ Franzmann PD Unification of the genera *Deleya* (Baumann et al. 1983), *Halomonas* (Vreeland et al. 1980), and *Halovibrio* (Fendrich 1988) and the species *Paracoccus halodenitrificans* (Robinson and Gibbons 1952) into a single genus, *Halomonas*, and placement of the genus *Zymobacter* in the family *Halomonadaceae*. Int J Syst Bacteriol 1996; 46:550-558 10.1099/00207713-46-2-550

[30] Ntougias S Zervakis GI Fasseas C *Halotalea alkalilenta* gen. nov., sp. nov., a novel osmotolerant and alkalitolerant bacterium from alkaline olive mill wastes, and emended description of the family *Halomonadaceae* Franzmann *et al* 1989, emend. Dobson and Franzmann 1996. Int J Syst Evol Microbiol 2007; 57:1975-1983 10.1099/ijs.0.65078-0 17766858

[31] Ben Ali Gam Z Abdelkafi S Casalot L Tholozan JL Oueslati R Labat M *Modicisalibacter tunisiensis* gen. nov., sp. nov., an aerobic, moderately halophilic bacterium isolated from an oilfield-water injection sample, and emended description of the family *Halomonadaceae* Franzmann et al. 1989 emend Dobson and Franzmann 1996 emend. Ntougias *et al* 2007. Int J Syst Evol Microbiol 2007; 57:2307-2313 10.1099/ijs.0.65088-0 17911302

[32] Arahal DR García MT Ludwig W Schleifer KH Ventosa A Transfer of *Halomonas canadensis* and *Halomonas israelensis* to the genus *Chromohalobacter* as *Chromohalobacter canadensis* comb. nov. and *Chromohalobacter israelensis* comb. nov. Int J Syst Evol Microbiol 2001; 51:1443-1448 10.1099/00207713-51-4-1443 11491344

[33] Cánovas D Vargas C Csonka LN Ventosa A Nieto JJ Synthesis of glycine betaine from exogenous choline in the moderately halophilic bacterium *Halomonas elongata* Appl Environ Microbiol 1998; 64:4095-4097 9758852 10.1128/aem.64.10.4095-4097.1998 PMC106611

[34] BAuA. 2010, Classification of bacteria and archaea in risk groups. TRBA 466, p. 56. www.baua.de

[35] Ashburner M Ball CA Blake JA Botstein D Butler H Cherry JM Davis AP Dolinski K Dwight SS Eppig JT Gene ontology: tool for the unification of biology. The Gene Ontology Consortium. Nat Genet 2000; 25:25-29 10.1038/75556 10802651 PMC3037419

[36] Csonka LN, O’Connor K, Larimer F, Richardson P, Lapidus A, Ewing AD, Goodner BW, Oren A. What we can deduce about metabolism in the moderate halophile *Chromohalobacter salexigens* from its genomic sequence. *In:* Gunde-Cimerman N, Oren A, Plemenitas A (*eds*). Adaptation to life at high salt concentrations in *Archaea*, *Bacteria*, and Eukarya. 2005. Springer, Dordrecht. pp. 267-285.

[37] Kushner DJ. Life in high salt and solute concentrations. *In:* Kushner DJ (*ed*) *Microbial Life in Extreme Environments.* London, Academic Press, 1978. pp. 317–368.

[38] O'Connor K Csonka LN The high salt requirement of the moderate halophile *Chromohalobacter salexigens* DSM3042 can be met not only by NaCl but by other ions. Appl Environ Microbiol 2003; 69:6334-6336 10.1128/AEM.69.10.6334-6336.2003 PMC201248 14532102

[39] O'Connor KA D R Zusman DR. Genetic analysis of tag mutants of *Myxococcus xanthus* provides evidence for two developmental aggregation systems. J Bacteriol 1990; 172:3868-3878 10.1128/jb.172.7.3868-3878.1990 PMC213368 2163391

[40] The DOE Joint Genome Institute www.jgi.doe.gov

[41] Phrap and Phred for Windows. MacOS, Linux, and Unix. www.phrap.com

[42] Sims D Brettin T Detter JC Han C Lapidus A Copeland A Glavina Del Rio T Nolan M Chen F Lucas S Complete genome sequence of *Kytococcus sedentarius* type strain (541^T^). Stand Genomic Sci 2009; 1:12-20 10.4056/sigs.761 PMC3035214 21304632

[43] Delcher AL Bratke K Powers E Salzberg S Identifying bacterial genes and endosymbiont DNA with Glimmer. Bioinformatics 2007; 23:673-679 10.1093/bioinformatics/btm009 17237039 PMC2387122

[44] Badger JH Olsen GJ CRITICA: Coding region identification tool invoking comparative analysis. Mol Biol Evol 1999; 16:512-524 10331277 10.1093/oxfordjournals.molbev.a026133

[45] Lowe TM Eddy SR tRNAscan-SE: a program for improved detection of transfer RNA genes in genomic sequence. Nucleic Acids Res 1997; 25:955-964 10.1093/nar/25.5.955 9023104 PMC146525

[46] Krogh A Larsson B von Heijne G Sonnhammer ELL Predicting transmembrane protein topology with a hidden Markov model: Application to complete genomes. J Mol Biol 2001; 305:567-580 10.1006/jmbi.2000.4315 11152613

[47] Bendtsen JD Nielsen H von Heijne G Brunak S Improved prediction of signal peptides: SignalP 3.0. J Mol Biol 2004; 340:783-795 10.1016/j.jmb.2004.05.028 15223320

[48] Oren A Larimer F Richardson P Lapidus A Csonka LN How to be moderately halophilic with broad salt tolerance: clues from the genome of *Chromohalobacter salexigens* Extremophiles 2005; 9:275-279 10.1007/s00792-005-0442-7 15902510

[49] Krejcík Z Hollemeyer K Smits TH Cook AM Isethionate formation from taurine in *Chromohalobacter salexigens*: purification of sulfoacetaldehyde reductase. Microbiology 2010; 156:1547-1555 10.1099/mic.0.036699-0 20133363

